# 

**Published:** 1834-01-01

**Authors:** 


### MEDICO-CHIRURGICAL REVIEW,

AND

### JOURNAL

OF

### PRACTICAL MEDICINE.

*(NEW SERIES.)*

VOLUME TWENTY,

[1st of OCTOBER, 1833, to 31st of MARCH,]

1834.

VOL. XXIV. of ANALYTICAL SERIES.

EDITED

BY JAMES JOHNSON, M.D.

PHYSICIAN EXTRAORDINARY TO THE KING,

AND

HENRY JAMES JOHNSON, M.R.C.S.

LATE HOUSE SURGEON TO ST. GEORGE'S AND THE LOCK HOSPITALS.

Surgeon Gen'l's Office LIBRARY
57459

LONDON:

PUBLISHED BY S. HIGHLEY, 32, FLEET STREET,

AND WEBB STREET, ST. THOMAS'S HOSPITAL.

*Re-printed in New York, by Mr. Wood.*

1834.

W1
ME 75 T

PRINTED BY G. HAYDEN,

Little College Street, Westminster.

### TO CORRESPONDENTS.

The analysis of the work on Cutaneous Diseases received from our Correspondent in L——l. must remain till next Number, when it will appear.

The Clinical Report from the Roachdale Dispensary is received.

The excellent analysis drawn up by Dr. Lucas, of Malta, has come safe to hand, and will be appropriated in our next.

SCOTO-BRITANNICUS. The Index of the 20 volumes, New Series, will not be incorporated with that of the four annual volumes of a preceding series.

Dr. CUMMING. The communication respecting the fatal case of Mœlena came safe to hand.

